# Supplementary material for: Plasma Proteomic Analysis Identified Proteins Associated with Faulty Neutrophils Functionality in Decompensated Cirrhosis Patients with Sepsis
Source: Cells. 2022 May 25;11(11):1745. doi: 10.3390/cells11111745 (PMC9179303; doi:10.3390/cells11111745)

Plasma proteomic analysis identified proteins associated with faulty neutrophils functionality in decompensated cirrhosis patients with sepsis

Rashi Sehgal[1,2], Navkiran Kaur[2], Rakhi Maiwall[3], Gayatri Ramakrishna[1], Jaswinder Singh Maras[1*], Nirupma Trehanpati[1*]

SUPPLEMENTARY INFORMATION

Corresponding Authors:

*Corresponding Authors:

Dr. Nirupma Trehanpati
Professor, Department of Molecular and Cellular Medicine
Institute of Liver and Biliary Sciences
D-1 Vasant Kunj, New Delhi 110070, India
trehanpati@ilbs.in, trehanpati@gmail.com
ORCID: 0000-0002-6109-0033

Dr. Jaswinder Singh Maras
Assistant Professor, Department of Molecular and Cellular Medicine
Institute of Liver and Biliary Sciences
D-1 Vasant Kunj, New Delhi 110070, India
jassi2param@gmail.com

SUPPLEMENTARY TABLE:

Supplementary Table S1: Lowest detection limit of cytokines, chemokines, and growth factors in cytokine bead array assay.

S. No.	Name	Lowest detection limit (pg/ml)
1	IL-6	10
2	IL-2	5.71
3	IL-4	9.99
4	IL-10	1.70
5	TNF-α	8.59
6	IFN- γ	15
7	GMCSF	15
8	MIF	0.21
9	IL-8	2.76
10	MCP-1	3.93
11	IP-10	2.03
12	GCSF	13
13	IL-1β	1.99
14	IL-18	17
15	TGF-b1	3
16	MIP-1A	2.04
17	MIP-1B	5.64
18	MIP-3A	4.44
19	ITAC	3.74
20	FRACTALKINE	1.25
21	ENA78	6.59
22	MCSF	14
23	LEPTIN	16
24	VEGF-A	5.37
25	MMP12	5.76
26	E SELECTIN	206
27	Angiopoietin-1	32
28	Eotaxin	0.62
29	HGF	6.49
30	IL-1RA	43
31	IL-12p40	1.35
32	IL-17A	2.27
33	IL-27	17
34	IL-33	23
35	MMP-1	8.52
36	MMP-13	7.96
37	MMP-7	11
38	MMP-8	36
39	P-SELECTIN	1348
40	TPO	94
41	TREM-1	689

Supplementary Table S2: Correlation of upregulated and downregulated proteins with the neutrophils, its oxidative burst activity, MELD, MELD Na and Bilirubin. R-value and p-value given in each cell. Cell containing no value shows no significant correlation.

	DNAJC13	GPI	GASTP1	PNP	ANPEP	COTL1	PGMA1	APOA1	PROS1	SERPINA3	MPO	MMRN1
NEUTROPHILS	0.945<br>0.002	0.994<br><0.0001	0.879<br>0.021	0.885<br>0.019	0.915<br>0.010	0.977<br><0.0001	0.905<br>0.013	-0.896<br>0.015	0.970<br>0.001	0.927<br>0.007	0.903<br>0.013	0.882<br>0.020
$CD11b^+$ NEUTRO	0.835<br>0.038	-	-	-	0.840<br>0.036	0.869<br>0.024	0.856<br>0.039	-	0.933<br>0.006	0.864<br>0.026	0.879<br>0.020	-
OXIDATIVE BURST	0.746<br>0.050	-	-	-	-	0.824<br>0.043	0.899<br>0.014	-	0.944<br>0.004	0.884<br>0.019	-	-
MELD	0.916<br>0.010	0.967<br>0.001	0.816<br>0.047	0.794<br>0.050	0.920<br>0.001	0.906<br>0.012	0.809<br>0.050	-0.836<br>0.037	0.906<br>0.013	0.828<br>0.041	0.957<br>0.002	-
MELD Na	0.953<br>0.003	0.980<br><0.0001	0.881<br>0.020	0.870<br>0.024	0.934<br>0.006	0.958<br>0.002	0.855<br>0.029	-0.881<br>0.020	0.936<br>0.006	0.880<br>0.020	0.938<br>0.005	0.838<br>0.037
Bilirubin	0.976<br><0.0001	0.972<br>0.001	0.933<br>0.006	0.908<br>0.012	0.966<br>0.001	0.987<br><0.0001	0.834<br>0.039	-0.863<br>0.026	0.921<br>0.009	0.895<br>0.015	0.915<br>0.010	0.868<br>0.002

Supplementary Table S3: Correlation of neutrophils, its phagocytic and oxidative burst activity with the cytokines, chemokine and growth factors. R-value and p-value given in each cell. Cell containing no value shows no significant correlation.

	IL-10	TPO	MIP-1α	IL-33	MCSF	MCP1	HGF	ENA78	MIP3α	MMP1	PSELECTIN	TREM1	IL-18	IL-4	IFN-γ	TNF-α	MMP7	VEGF-A	IL-12p40	IL-1β	TGF-β1	MIP-1β	ESELECTIN	MIF	IL-27	EOTAXIN	IL-17A	IL-2	IP-10
NEUTROPHILS	-	-	-	0.746<br>0.027	-	0.800<br>0.050	0.867<br>0.025	0.808<br>0.050	0.902<br>0.014	0.875<br>0.022	0.878<br>0.021	0.957<br>0.003	0.980<br><0.0001	0.958<br>0.002	0.992<br><0.0001	0.966<br>0.002	0.959<br>0.002	0.972<br>0.001	0.967<br>0.001	0.915<br>0.010	0.956<br>0.003	0.834<br>0.039	-	0.891<br>0.017	0.932<br>0.006	0.936<br>0.005	0.939<br>0.005	0.969<br>0.001	0.973<br>0.001
CD11b$^+$ NEUTRO	-	-	-	0.863<br>0.027	-	-	-	-	-	-	-	0.880<br>0.020	0.963<br>0.015	0.855<br>0.029	0.914<br>0.011	0.851<br>0.032	0.873<br>0.023	0.859<br>0.028	0.851<br>0.031	0.833<br>0.039	0.958<br>0.026	0.857<br>0.029	-	0.844<br>0.034	0.947<br>0.004	0.939<br>0.005	0.879<br>0.021	0.932<br>0.006	0.960<br>0.002
CD11b$^+$CXCR1$^+$	0.865<br>0.026	-	-		0.862<br>0.027	-	-	-	-	-	-	-	-	-	-	-	-	-	-	-	-	-	-	-	-	-	-	-	-
PHAGOCYTIC ACTIVITY	-	-0.915<br>0.010	-0.913<br>0.010	-0.862<br>0.027	-	-	-	-	-	-	-	-	-	-	-	-	-0.816<br>0.047	-	-	-	-	-0.813<br>0.048	-0.832<br>0.039	-0.831<br>0.040	-0.877<br>0.022	-0.873<br>0.023	-0.874<br>0.023	-0.823<br>0.044	-
OXIDATIVE BURST	-	-	-	-	-	-	-	-	-	-	-	-	0.903<br>0.042	0.849<br>0.032	0.883<br>0.019	0.821<br>0.045	0.811<br>0.050	0.796<br>0.050	-	0.863<br>0.027	0.977<br>0.000	-	-	-	0.934<br>0.006	0.893<br>0.016	0.868<br>0.025	0.924<br>0.008	0.959<br>0.002

Supplementary Table S4: Correlation of upregulated and downregulated proteins with the cytokines, chemokine and growth factors. R-value and p-value given in each cell. Cell containing no value shows no significant correlation.

	MIP-1α	ANGIOPOIENTIN	MCSF	IL-8	MMP8	MCP1	HGF	ENA78	MIP3a	MMP1	PSELECTIN	TREM1	IL-18	IL-4	IFN-γ	TNF-α	MMP7	VEGF-A	IL-12p40	IL-1β	TGF-β1	MIP-1β	ESELECTIN	MIF	IL-27	EOTAXIN	IL-17A	IL-2	IP-10
DNAJC13	-	-	-	0.837<br>0.037	-	0.943<br>0.005	0.973<br>0.001	0.899<br>0.014	0.968<br>0.001	0.934<br>0.006	0.961<br>0.002	0.945<br>0.004	0.926<br>0.008	0.906<br>0.013	0.944<br>0.004	0.947<br>0.004	0.981<br><0.0001	0.985<br><0.0001	0.986<br><0.0001	0.807<br>0.050	0.823<br>0.044	0.858<br>0.028	0.871<br>0.023	0.947<br>0.004	0.854<br>0.030	0.900<br>0.014	0.899<br>0.015	0.900<br>0.014	0.874<br>0.022
GPI	-	-	-	-	-	-	0.835<br>0.038	-	0.874<br>0.023	0.848<br>0.033	0.834<br>0.038	0.960<br>0.002	0.987<br><0.0001	0.943<br>0.005	0.984<br><0.0001	0.945<br>0.004	0.923<br>0.008	0.950<br>0.003	0.939<br>0.005	0.916<br>0.010	0.959<br>0.002	0.804<br>0.050	-	0.850<br>0.032	0.912<br>0.011	0.910<br>0.011	0.905<br>0.013	0.950<br>0.004	0.965<br>0.002
GSTP1	-	-	0.839<br>0.036	0.924<br>0.008	0.885<br>0.019	0.0049	0.983<br><0.0001	0.959<br>0.002	0.985<br><0.0001	0.966<br>0.001	0.992<br><0.0001	0.895<br>0.016	0.839<br>0.036	0.885<br>0.019	0.899<br>0.014	0.931<br>0.007	0.947<br>0.004	0.964<br>0.002	0.968<br>0.001	-	-	-	0.802<br>0.050	0.893<br>0.016	-	0.813<br>0.049	0.857<br>0.029	0.838<br>0.037	-
PNP	-	0.824<br>0.043	-	0.906<br>0.013	0.860<br>0.028	0.884<br>0.019	0.925<br>0.008	0.985<br><0.0001	0.965<br>0.002	0.957<br>0.002	0.994<br>0.000	0.849<br>0.032	0.809<br>0.050	0.932<br>0.007	0.913<br>0.010	0.959<br>0.002	0.953<br>0.003	0.956<br>0.003	0.971<br>0.001	0.845<br>0.034	-	-	-	0.863<br>0.026	0.806<br>0.050	0.826<br>0.042	0.916<br>0.010	0.888<br>0.018	0.842<br>0.035
ANPEP	-	-	0.937<br>0.005	-	-	0.886<br>0.018	0.952<br>0.003	-	0.917<br>0.009	0.875<br>0.022	0.861<br>0.027	0.978<br><0.0001	0.952<br>0.003	0.819<br>0.046	0.901<br>0.014	0.866<br>0.025	0.895<br>0.015	0.937<br>0.006	0.918<br>0.009	-	-	0.835<br>0.038	0.824<br>0.043	0.890<br>0.017	-	0.830<br>0.040	-	0.805<br>0.050	0.803<br>0.050
COTL1	-	-	-	-	-	0.821<br>0.045	0.921<br>0.009	0.899<br>0.015	0.967<br>0.001	0.956<br>0.003	0.945<br>0.004	0.963<br>0.002	0.942<br>0.005	0.978<br><0.0001	0.993<br><0.0001	0.992<br><0.0001	0.954<br>0.003	0.994<br><0.0001	0.990<br><0.0001	0.930<br>0.007	0.904<br>0.013	-	-	0.856<br>0.029	0.858<br>0.028	0.868<br>0.024	0.918<br>0.009	0.935<br>0.006	0.925<br>0.008
GCA	-	-	0.797<br>0.050	0.977<br><0.0001	0.938<br>0.005	0.946<br>0.004	0.954<br>0.003	0.960<br>0.002	0.945<br>0.004	0.926<br>0.008	0.975<br><0.0001	0.799<br>0.050		0.808<br>0.050	0.810<br>0.050	0.864<br>0.026	0.901<br>0.014	0.900<br>0.014	0.913<br>0.010	-	-	-	-	0.861<br>0.028	-	-	0.803<br>0.050	-	-
PGAM1	-	-	-	-	-	-	-	-	-	-	-	-	0.819<br>0.045	0.960<br>0.002	0.923<br>0.008	0.926<br>0.008	0.849<br>0.032	0.856<br>0.029	0.866<br>0.026	0.979<br>0.000	0.967<br>0.001	-	-	-	0.889<br>0.017	0.829<br>0.041	0.937<br>0.006	0.952<br>0.003	0.955<br>0.003
APOA1	-	-	-	-	-	-0.831<br>0.040	-0.814<br>0.048	-0.903<br>0.013	-0.863<br>0.026	-0.841<br>0.036	-0.917<br>0.009	-	-0.807<br>0.050	-0.924<br>0.008	-0.900<br>0.014	-0.934<br>0.006	-0.963<br>0.002	-0.911<br>0.011	-0.937<br>0.006	-0.849<br>0.032	-0.872<br>0.023	-	-0.813<br>0.049	-0.891<br>0.017	-0.921<br>0.009	-0.917<br>0.009	-0.985<br>0.000	-0.955<br>0.003	-0.917<br>0.009
AHSG	-	0.875<br>0.022	-	-	-	-	-	0.841<br>0.035	-	-	0.799<br>0.050	-		0.898<br>0.015	0.810<br>0.050	0.868<br>0.025	-	-	0.805<br>0.050	0.896<br>0.015	0.831<br>0.040	-	-	-	-	-	0.903<br>0.013	0.870<br>0.024	0.839<br>0.036
PROS1	-	-	-	-	-	-	-	-	0.837<br>0.037	0.829<br>0.041	0.819<br>0.046	0.891<br>0.017	0.924<br>0.008	0.974<br>0.001	0.975<br>0.000	0.954<br>0.003	0.897<br>0.015	0.919<br>0.009	0.918<br>0.009	0.975<br>0.009	0.989<br><0.0001	-	-	-	0.916<br>0.010	0.882<br>0.019	0.936<br>0.005	0.970<br>0.001	0.982<br><0.0001
SERPINA3	-	0.841<br>0.036	-	-	-	-	-		0.835<br>0.038	0.848<br>0.032	0.798<br>0.050	0.883<br>0.019	0.881<br>0.020	0.963<br>0.002	0.954<br>0.003	0.937<br>0.005	0.824<br>0.043	0.893<br>0.016	0.882<br>0.019	0.990<br>0.000	0.942<br>0.005	-	-	-	0.812<br>0.049	-	0.860<br>0.027	0.903<br>0.014	0.920<br>0.009
MPO	0.815<br>0.482	-	0.831<br>0.040	-	-	0.927<br>0.007	0.888<br>0.018	-	0.832<br>0.039	-	0.817<br>0.047	0.893<br>0.016	0.929<br>0.007	-	0.859<br>0.028	0.823<br>0.044	0.933<br>0.006	0.894<br>0.016	0.892<br>0.017	-	-	0.971<br>0.001	0.963<br>0.002	0.981<br><0.0001	0.875<br>0.022	0.938<br>0.005	0.835<br>0.038	0.852<br>0.031	0.842<br>0.035
MMRN1	-	0.926<br>0.007	-	-	-	-	-	0.943<br>0.004	0.905<br>0.013	0.921<br>0.008	0.930<br>0.007	-	-	0.976<br>0.000	0.925<br>0.008	0.969<br>0.001	0.893<br>0.016	0.918<br>0.009	0.931<br>0.007	0.949<br>0.004	0.864<br>0.026	-	-	-	0.807<br>0.050	-	0.926<br>0.007	0.910<br>0.011	0.881<br>0.020

Supplementary Table S5: Sequence of primers used for RT-PCR

Name	Forward primer	Reverse primer
**18s**	AAGTACGCACGGCCGGTACA	AGCGCCCGTCGGCATGTATTA
**DNAJC13**	AGTGGCAAAGACTAGCTGGAA	TGTCCTCTTTTTGAGCAATGCC
**TMSB4X**	ACTGCCTTCCAAAGAAACGAT	AGGGGCAGCACAGTCATTTA
**AHSG**	CATGGCCCAGGGCTGATTTA	CTGCTGAGGCCACACCTTTA
**HMGB1**	ATA TGGCAAAAGCGGACAAG	AGGCCAGGATGTT CTCCTTT
**HIF-1α**	GTTTACTAAAGGACAAGTCACC	TTCTGTTTGTTGAAGGGAG
**ATG5**	TTGACGTTGGTAACTGACAAAGT	TGTGATGTTCCAAGGAAGAGC
**ATG12**	TAGAGCGAACACGAACCATCC	CACTGCCAAAACCATCATAGAGA

SUPPLEMENTARY FIGURES:

SUPPLEMENTARY FIGURE S1: Scatter dot plot shows the percent expression of Neutrophils, $CD11b^{+}CD66b^{+}$ cells, $CXCR1^{+}$ and $CXCR2^{+}$ on $CD11b^{+}CD66b^{+}$ neutrophils in sepsis and w/o sepsis at different time points i.e D0, D3 and D7.

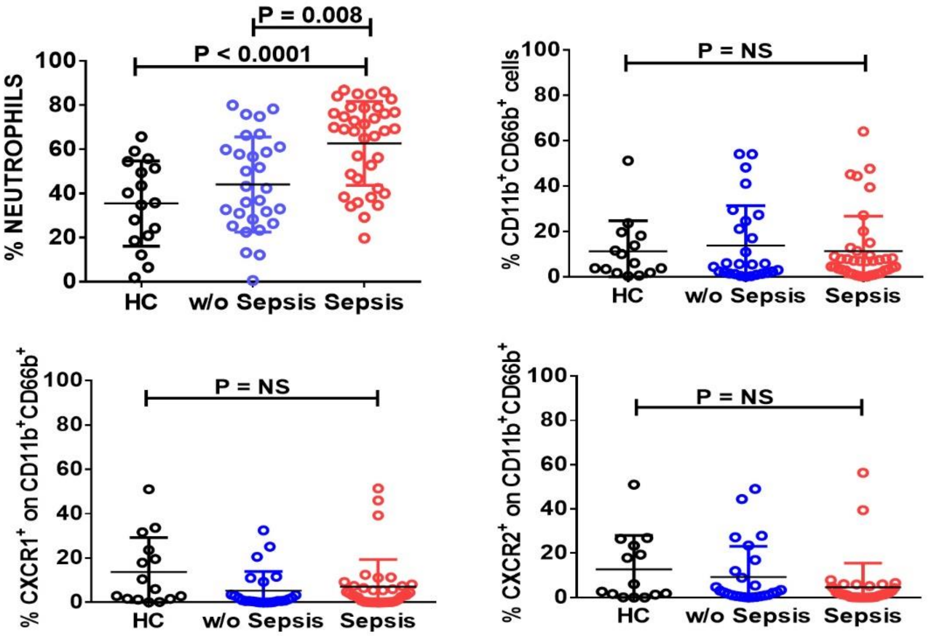

SUPPLEMENTARY FIGURE S2: Scatter dot plot shows the percent expression of Neutrophils, $CD11b^{+}CD66b^{+}$ cells, $CXCR1^{+}$ and $CXCR2^{+}$ on $CD11b^{+}CD66b^{+}$ neutrophils **A)** based on alcohol (ALC) and other etiologies (NASH and cryptogenic) in patients with or w/o sepsis; **B)** with or w/o AKI as a decompensating event in sepsis patients.

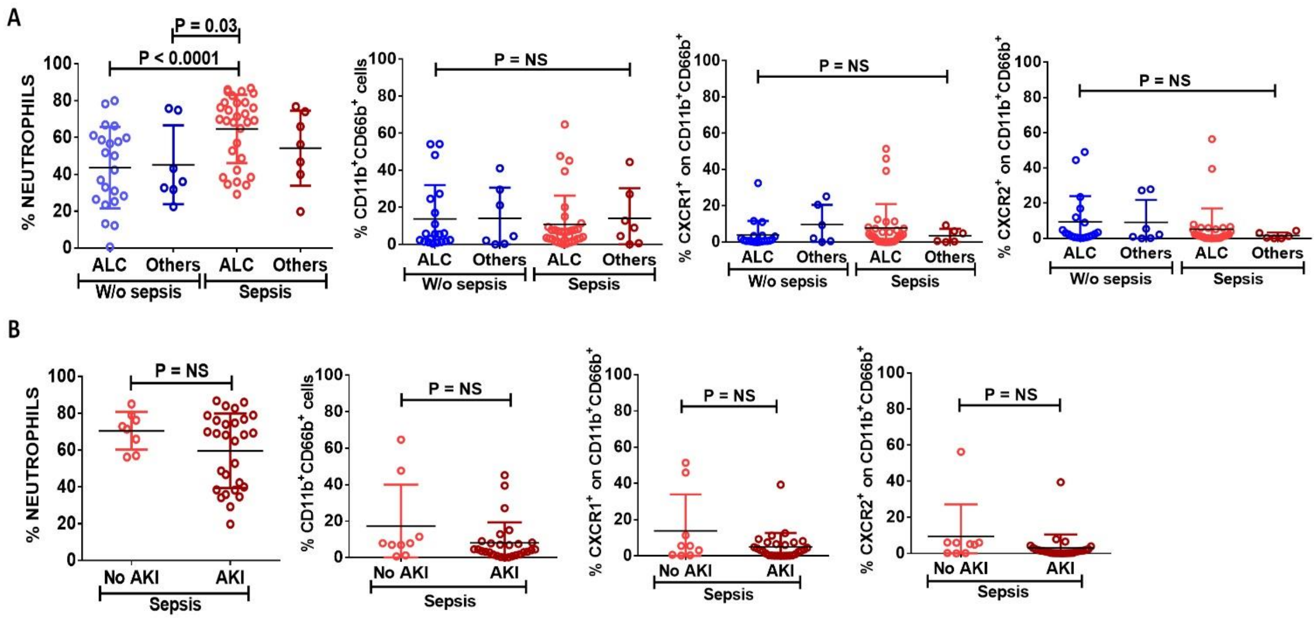

SUPPLEMENTARY FIGURE S3: Scatter dot plot shows the percent expression of Neutrophils, $CD11b^{+}CD66b^{+}$ cells, $CXCR1^{+}$ and $CXCR2^{+}$ on $CD11b^{+}CD66b^{+}$ neutrophils based on no resolution and resolution of sepsis upon similar treatment in decompensated cirrhosis with sepsis patients at D0, D3 and D7.

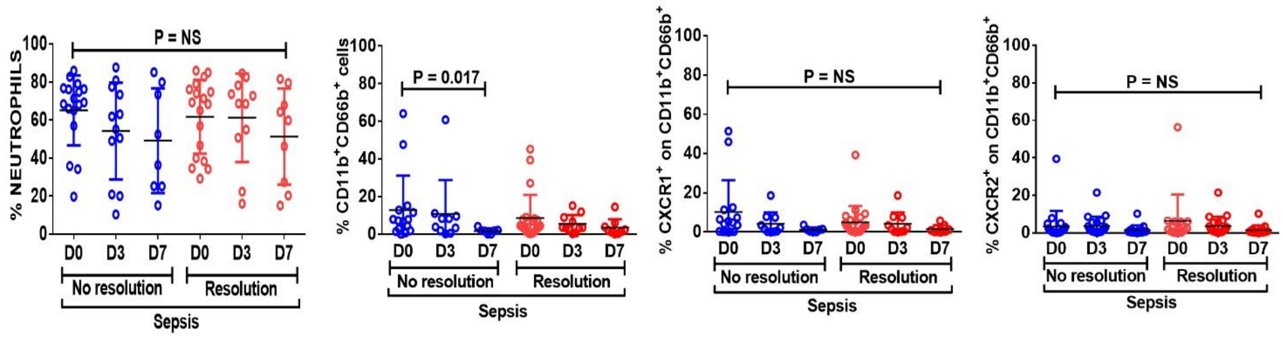

SUPPLEMENTARY FIGURE S4: Scatter dot plot shows the percent expression of phagocytic activity and oxidative burst activity (*E. coli*) of neutrophils in sepsis and w/o sepsis at D0, D3 and D7.

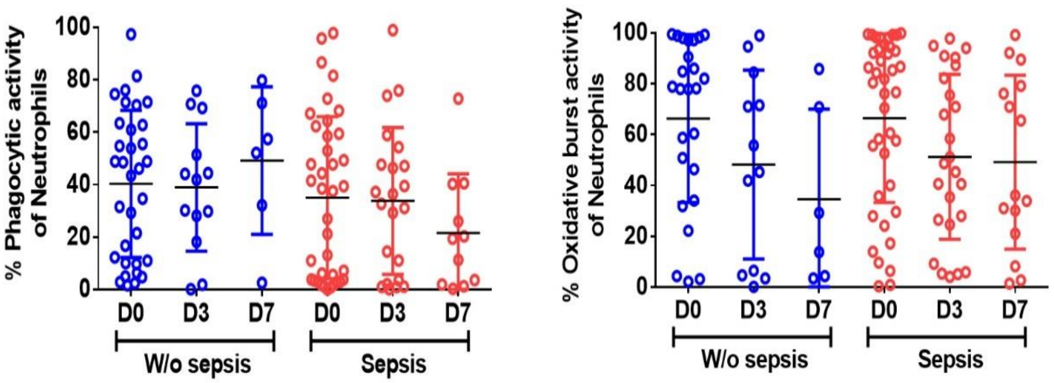

SUPPLEMENTARY FIGURE S5: Cell free DNA in sepsis and w/o sepsis at different time points i.e D0, D3 and D7.

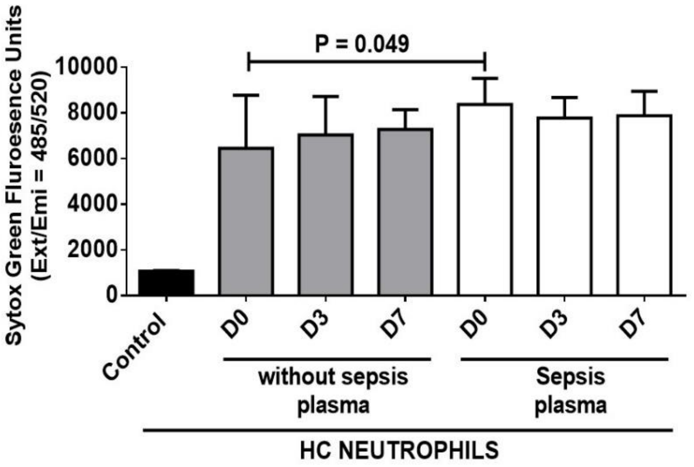

SUPPLEMENTARY FIGURE S6: Percent expression of **A)** HLA-DR on monocytes **B)** classical, intermediate, and non-classical monocytes **C)** HLA-DR on classical, intermediate, and non-classical monocytes, **D)** phagocytic and **E)** oxidative burst activity (E. coli) of monocytes in sepsis and w/o sepsis at different time points i.e D0, D3 and D7.

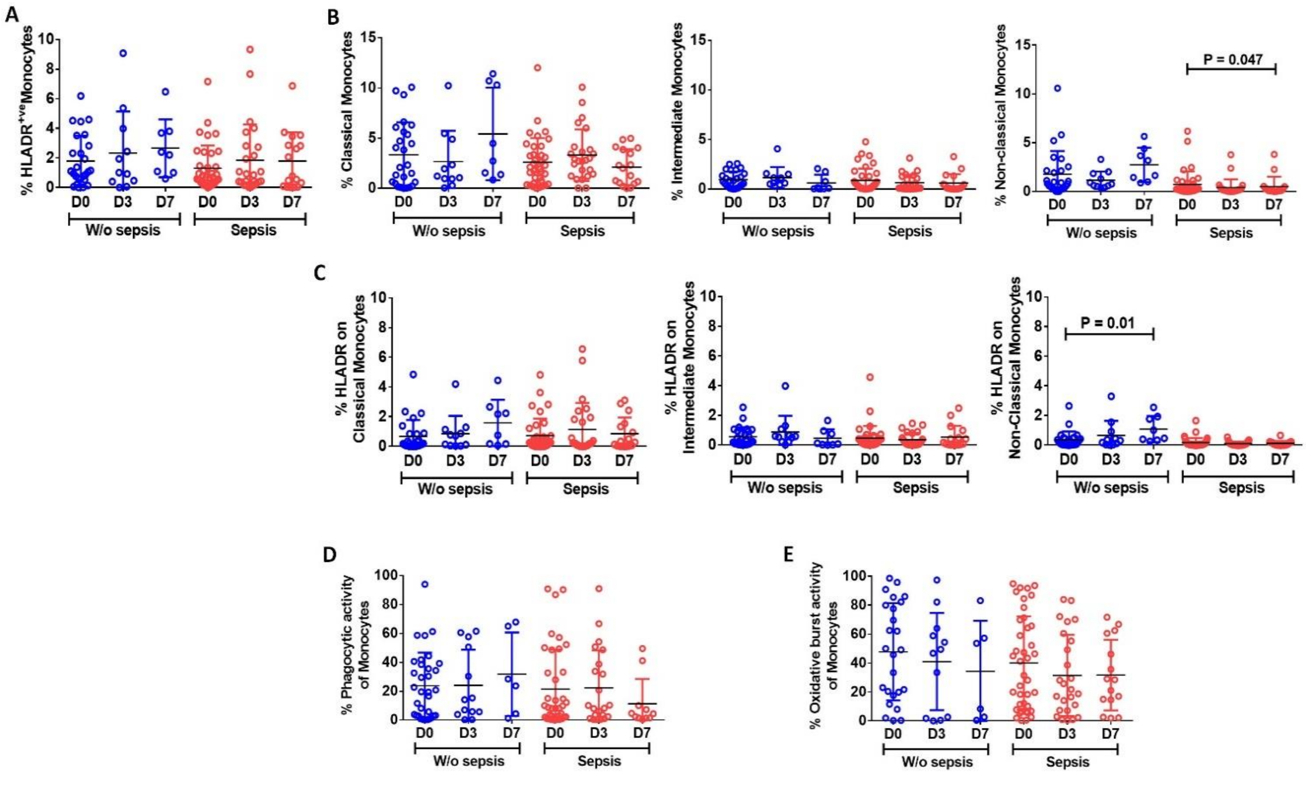

SUPPLEMENTARY FIGURE S7: Percent expression of **A)** HLA-DR on monocytes **B)** classical, intermediate, and non-classical monocytes based on no resolution and resolution of sepsis upon similar treatment in decompensated cirrhosis with sepsis patients at D0, D3 and D7.

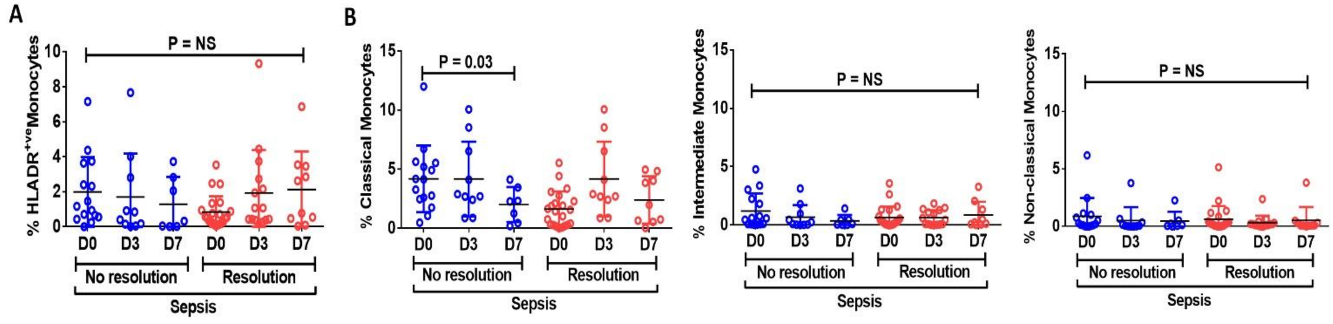

Supplement: Supplementary file 1 [file cells-11-01745-s001.zip › cells-1605084-supplementary.pdf]
